# Ashwagandha (Withania somnifera) as an adjunct in schizophrenia: clinical signals, safety, and research priorities

**DOI:** 10.1192/j.eurpsy.2026.12166

**Published:** 2026-07-31

**Authors:** B. Larisa, N. Igor

**Affiliations:** Department of Mental Health, Medical Psychology, and Psychotherapy, Nicolae Testemițanu State University of Medicine and Pharmacy, Chisinau, Moldova, Republic of

## Abstract

**Introduction:**

Negative and stress-related symptom domains in schizophrenia often respond suboptimally to antipsychotics. Standardized *Withania somnifera* root extracts show anti-inflammatory and stress-modulating activity in preclinical work and are being evaluated as adjunctive therapy.

**Objectives:**

To synthesize clinical efficacy and safety signals for WSE in schizophrenia and outline practical guardrails and research priorities.

**Methods:**

We conducted a narrative synthesis (PubMed, Scopus, Web of Science) of 56 publications (2012–Sept 2025). Selection criteria included randomized controlled trials (RCTs) in schizophrenia with PANSS assessments, as well as publications that examined mitochondrial endpoints (mtDNA, ATP, oxygen consumption/OXPHOS) and/or pathway markers (BDNF, SIRT1). Exclusion criteria were brand names, non-standardized blends, and non-English full texts.

**Results:**

We found only one 12-week, double-blind RCT (n=66) in which a standardized *Withania somnifera* root extract (1,000 mg/day) was added to stable antipsychotics during symptom exacerbation. Compared with placebo, the extract produced greater reductions in negative, general, and total PANSS scores, with no effect on positive symptoms. Inflammatory markers showed no between-group differences. Fewer participants required antipsychotic dose escalation. Tolerability was generally good, with mostly mild, transient gastrointestinal symptoms and somnolence; weight, vital signs, and laboratory values remained stable. Post-marketing reports describe rare idiosyncratic liver injury—typically cholestatic or mixed—with onset at approximately 2–12 weeks and recovery after discontinuation, as well as occasional thyroid-index shifts. No major drug–drug interactions are established. Use is not recommended during pregnancy or lactation.

**Conclusions:**

Preliminary evidence suggests that adjunctive WSE may improve negative and general symptom domains and reduce perceived stress in schizophrenia, without affecting positive symptoms. Preclinical data indicate that standardized root-only *W. somnifera* may enhance neuronal mitochondrial biogenesis and energetics via BDNF/TrkB/Akt and SIRT1 pathways (e.g., withanolide A, withanoside IV), consistent with stress–mitochondria phenotypes in psychosis. These findings remain exploratory, and WSE should not be used as monotherapy. Replication in adequately powered, multicenter RCTs with standardized extracts, biomarker panels, and prospective safety monitoring is required before clinical recommendations can be made.

**Keywords:**

schizophrenia, PANSS, adjunctive therapy, *Withania somnifera*, mitochondrial effects, pregnancy, lactation

**Disclosure of Interest:**

None Declared

